# Comparison of the Main Metabolites in Different Maturation Stages of *Camellia* *vietnamensis* Huang Seeds

**DOI:** 10.3390/molecules27206817

**Published:** 2022-10-12

**Authors:** Heqin Yan, Wei Zheng, Zhouchen Ye, Jing Yu, Yougen Wu

**Affiliations:** 1 Key Laboratory for Quality Regulation of Tropical Horticultural Plants of Hainan Province, College of Horticulture, Hainan University, Haikou 570228, China; 2 College of Tropical Crops, Hainan University, Haikou 570228, China

**Keywords:** *Camellia vietnamensis* Huang, UHPLC/Q-TOF-MS, maturation stages, metabolomics

## Abstract

*Camellia vietnamensis* Huang is an important woody oil crop in China, which has attracted much attention because of its abundant nutritional components and pharmaceutical value. Its seeds undergo a complex series of physiological and biochemical changes during maturation, with consequent alterations in metabolites. In order to investigate the endogenous metabolism of *C. vietnamensis* on Hainan Island during seed development, in this study, ultra-high-performance liquid tandem chromatography coupled with quadrupole time-of-flight mass spectrometry (UHPLC/Q-TOF-MS) and multivariate statistical analysis (MSA) were used to analyze the differences in the chemical compounds of *C. vietnamensis* seeds among the four maturation stages. A total of 293 metabolites were identified from the methanol extract of the seeds of *C. vietnamensis*. Five metabolites, belonging to benzene and substituted derivatives, 5′-deoxyribonucleosides and linear 1,3-diarylpropanoids, were found in all three comparison groups, with consistently down-regulated trends. The Kyoto Encyclopedia of Genes and Genomes (KEGG) results showed that phloretin and 5′-methylthioadenosine were the differentially expressed metabolites when seeds were in the growth periods of S2 and S3, and indole and L-tryptophan were the differentially expressed metabolites when seeds were in the growth periods of S3 and S4. In addition, 34 flavonoid metabolites were detected, of which 4 were differentially expressed. It was indicated that flavonoids dynamically change during all the oil-tea camellia seed development stages. The findings provide data for the better understanding of endogenous metabolic pathways during *C. vietnamensis* seed development.

## 1. Introduction

*Camellia* spp. have been distributed and cultivated in southern China for a long period, where they are mainly distributed in the Yangtze River Basin [1,2], and some other tropical countries—Thailand and Vietnam have some certain areas with planting [3]. *Camellia* oil is used not only as a healthy edible oil but also for cosmetics and medical objectives due to its beneficial ingredients, such as unsaturated fatty acids, triterpenes saponin, squalene, vitamin E, β-amyrin, stigmasterol, quercetin, and flavonoids [3,4]. Because of large-scale cultivation under different environmental conditions in China, *Camellia* spp. populations have developed different morphological characteristics, growth habits, and oil qualities [5]. *Camellia vietnamensis* Huang, a species of oil-tea *Camellia* trees from Hainan Island, belongs to the *Camellia* genus, Theaceae, and is regarded as an independent and a traditional plant resource according to a long period of geographic isolation from the mainland [6]. *C. vietnamensis* is somewhat different from the widely grown *C. oleifera* on the China mainland, mainly characterized by a large and thick fruit size, high oil content of the seed but low fruit yield, of which the oil is of excellent quality and has a unique scent and mouthfeel [7]. It is more suited to a tropical climate and has a large amount of genetic variation with a higher content of active ingredients in the oil [6,7,8].

Untargeted metabolomics provides comprehensive chemical profiling and plays a key role in quality control, chemical composition changes, and processing mechanism studies [9,10]. It has been widely used in plant classification [11], processing [12], chemical component changes [13,14], and differential metabolite analysis [10]. Compared with various profiling techniques in plant metabolomics, ultra-high-performance liquid tandem chromatography coupled with quadrupole time-of-flight mass spectrometry (UHPLC/Q-TOF-MS) has a higher sensitivity and separation of metabolites, and it is widely used in revealing the endogenous metabolism of plants. A UHPLC/Q-TOF-MS/MS-guided approach has been used to isolate, purify, and analyze the sulfur-containing derivatives from sulfur-fumigated ginseng [15]. Lee et al. used soybean (*Glycine max* (L.) Merr.) seeds as experimental materials to study the metabolite levels of seeds at different maturity stages [16]. They found that oil and total isoflavones were highly correlated with seed maturity. In addition, they also found that there were differences in the accumulation of bioactive secondary metabolites, such as anthocyanins and isoflavones, during the mature period of the seeds. Therefore, in order to maximize the effectiveness of these metabolites, it is necessary to avoid harvesting seeds in immature or over mature seasons. Liu et al. found that there was a significant difference in the fatty acid content of *Plukenetia volubilis* seeds at different growth stages [17]. In addition, the unsaturated fatty acid content increased sharply in the mature stage of the seeds, especially α-linolenic acid and linoleic acid, providing a reference for the rational development and utilization of these seeds and the extraction of certain kinds of fatty acids. However, the chemical composition of seeds during different developmental periods has seldom been studied in *C. vietnamensis*. Therefore, it is meaningful and feasible to compare the main metabolites in different maturation stages of *C. vietnamensis* seeds using UHPLC/Q-TOF-MS.

At present, numerous studies have focused on the key metabolites associated with the high oil quality and disease prevention of oil-tea trees using multi-omics analysis [6,18], as well as sugar metabolism and the transporters of sugar during seed development [19]. However, changes in the composition and content of nutrient components during the seed development of *C. vietnamensis* are still unclear. Hence, this study examined the principal chemical constituents of *Camellia* seeds in four different developmental periods using UHPLC/Q-TOF-MS coupled with multivariate statistical analysis, and subsequently the different metabolites were elucidated by the Kyoto Encyclopedia of Genes and Genomes (KEGG). The findings were intended to provide theoretical support for analyzing the endogenous metabolism of *C. vietnamensis* during its seed development and for elucidating the mechanisms of camellia oil quality formation, as well as to provide chemical information for the potential health benefits of *C. vietnamensis*.

## 2. Results

### 2.1. Metabolite Detection in Seeds from C. vietnamensis at Different Maturation Stages

*C. vietnamensis* seeds appear from August and then gradually expand to big fruits with thin skin. Seed extracts derived from four maturation stages of *C. vietnamensis* were profiled by UHPLC/Q-TOF-MS to examine the plant’s response to the accumulation of nutrition and the development of metabolites or derivatives. A total of 293 metabolites were detected in the seeds. In all the four stages, UHPLC/Q-TOF-MS analysis data identified classes including cinnamaldehydes (2), fatty acyls (5), flavonoids (22), isoflavonoids (2), phenols (1), and steroids and steroid derivatives (6) (Table S1).

### 2.2. Multivariate Analysis of Seeds in C. vietnamensis

**Figure 1:**
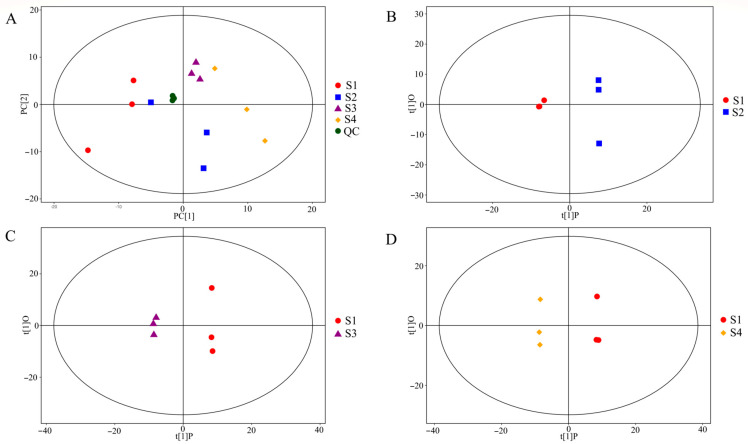
Multivariate analysis of seeds of *C. vietnamensis*. (**A**) PCA analysis of metabolic profiles from four sample groups and QC. (**B**–**D**) OPLS-DA models of seeds of *C. vietnamensis* at different maturity stages in positive and negative ion modes.

UHPLC/Q-TOF-MS data profiles showed an overlapped overview of response intensity and retention time (rtime) of the peak, indicating minor variations due to instrument error (Figure S2). Quantitative data for the total sample with quality control (QC) were subjected to PCA analysis to investigate variation in the metabolic profiles of Stages S1-S4 of *C. vietnamensis* seeds. The results in two-dimensional space showed that the QC samples were clustered closely, and all the samples were within the 95% confidence interval (Hotelling’s T-squared ellipse), which indicated the good repeatability of this experiment (Figure 1A). As manifested in Figure 1A, the samples of S3 and S4 were evidently categorized and assembled separately on the scorer plot, which suggested that the *C. vietnamensis* seeds from the two mature stages could be noticeably detached from S1 and S2, while the samples from S1 and S2 were too discrete, and the classification was not obvious. In conclusion, the metabolites of *C. vietnamensis* seeds from the four maturation stages could not be differentiated through the PCA algorithm. Consequently, the exploration of differential metabolites among the four stages were further studied by the OPLS-DA approach. OPLS-DA was modeled to analyze the metabolic profiling for further distinguishing the differences between the S1–S4 groups and determining the differential metabolites of *C. vietnamensis* seeds at different maturation stages (Figure 1B–D and Figure S3). It showed that the established model had no over-fitting, the sample discrimination was quite distinct, and all the samples were within the 95% confidence interval, which could explain the significant difference in the metabolites between different groups.

### 2.3. Global Trend Analysis of Metabolites during Seed Maturation in C. vietnamensis

**Figure 2:**
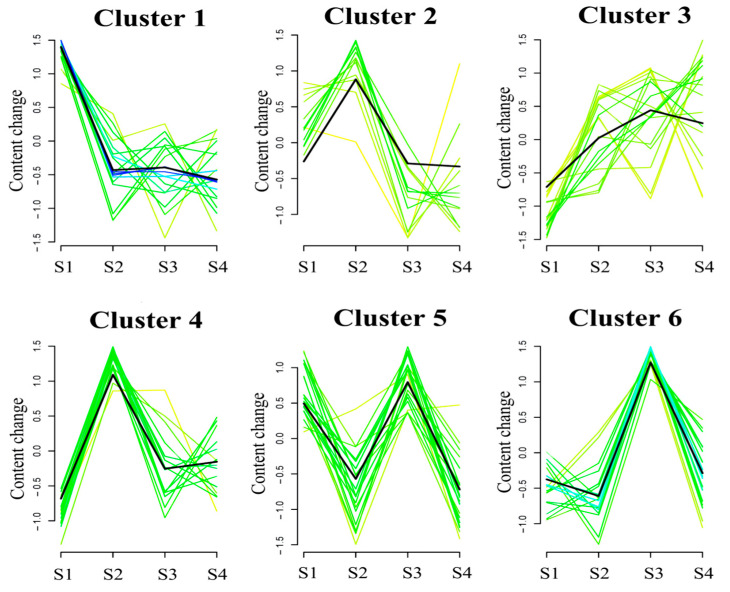
Cluster analysis of metebolites of seeds of *C. vietnamensis* in four maturation stages by using the Mfuzz package. Cluster 1: metabolites were mainly accumulated in S1; Clusters 2 and 4: metabolites were mainly accumulated in S2; Clusters 3, 5, and 6: metabolites were mainly accumulated in S3.

To analyze changes during *C. vietnamensis* maturation, we clustered and classified the total detected metabolites. The metabolites were grouped into six clusters that could conclude the change trend of the metabolite content of each cluster (Figure 2). The metabolites in Cluster 1 were strongly detected in S1, mainly flavonoids, such as phloretin, 2-(3,4-dihydroxyphenyl)-3,5,7-trihydroxy-3,4-dihydro-2H-1-benzopyran-4-one, 5,7-dihydroxy-2-(4-hydroxyphenyl)-3,4-dihydro-2H-1-benzopyran-4-one, and peonidin-3-glucoside. The metabolites in Clusters 2 and 4 were quite accumulated in S2, mainly terpenoids, prenol lipids, and other substances, such as coumarin, β-amyrin, caryophyllene alpha-oxide, alpha-amyrone, Olean-12-en-28-oic acid, 3-hydroxy-, (3beta,5xi,9xi,18xi)-, and so on. The metabolites in Clusters 3, 5, and 6 had the highest accumulation in S3, mainly glycosides, flavonoids, and other substances, such as brassicoside, kaempferol 3-glucosyl-(1- > 4)-rhamnosyl-(1- > 2)-glucoside, panasenoside, naringenin-7-O-glucoside, cyanidin 3-glucoside, luteolin 7-glucoside, (+)-Epicatechin, quercitrin, and so on. Notably, flavonoids, tannins, phenols, and mini peptides in Cluster 1 showed drops in concentration during *C. vietnamensis* maturation (S2–S4) (Figure 2 and Table S2).

### 2.4. Identification of Differential Metabolites

**Figure 3:**
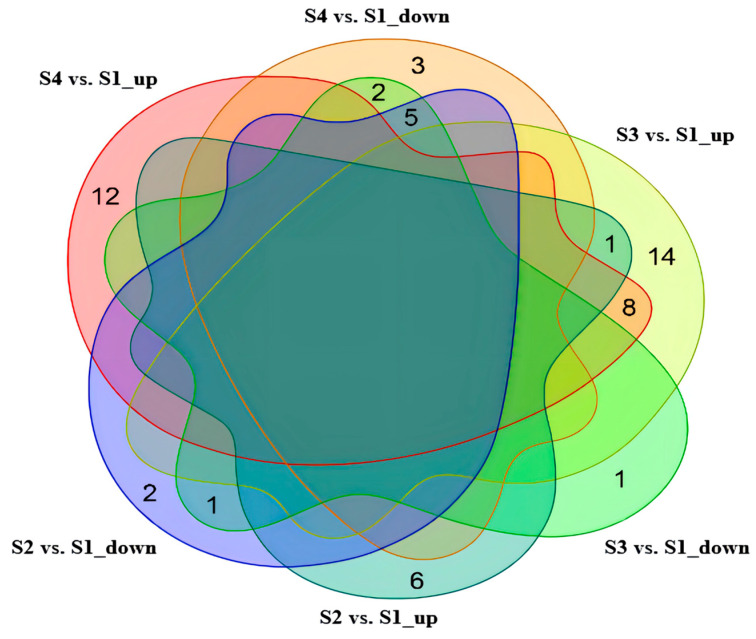
Expression analysis of differential metebolites from seeds of *C. vietnamensis* during different maturation stages.

**Figure 4:**
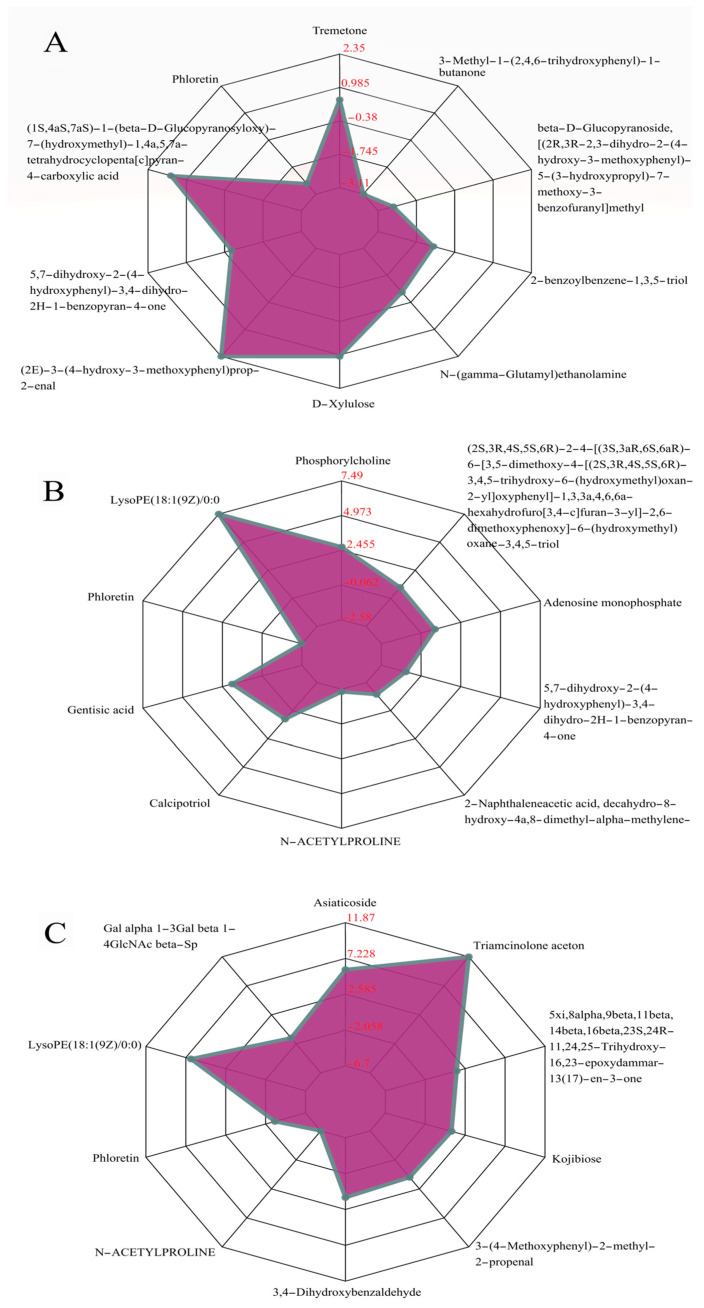
Radar plots of differential metabolites from seeds of *C. vietnamensis* during different maturation stages. Red fonts in the figures represented the corresponding ratios which were calculated for the quantitative values of the metabolites, and the logarithmic transformation was taken as the base 2. (**A**) S1 vs. S2; (**B**) S1 vs. S3; (**C**) S1 vs. S4.

Based on the condition of a variable importance in projection (VIP) of >1 of the first principal component in the OPLS-DA model and a *p*-value (Student’s *t* test) of univariate analysis of <0.05, the differential metabolites were screened (Table S3). Furthermore, 15, 32, and 30 metabolites were screened in S1 vs. S2, S1 vs. S3, and S1 vs. S4, respectively (Figure 3). We also found that the up-regulated metabolites with the largest fold change among the three comparison groups were triamcinolone acetone, lysoPE(18:1(9Z)/0:0), asiaticoside, and hydrocinnamic acid, and the down-regulated metabolites were N-acetylproline, 3-methyl-1-(2,4,6-trihydroxyphenyl)-1-butanone, and phloretin (Figure 3 and Table S3). In addition, five metabolites, belonging to benzene and substituted derivatives, 5′-deoxyribonucleosides and linear 1,3-diarylpropanoids, were found in all three comparison groups, with the consistent down-regulated trends. For each comparison group, this study further used the radar plots to display the trend change of the corresponding content of these metabolites (Figure 4).

### 2.5. Analysis of Differential KEGG Pathway

**Figure 5:**
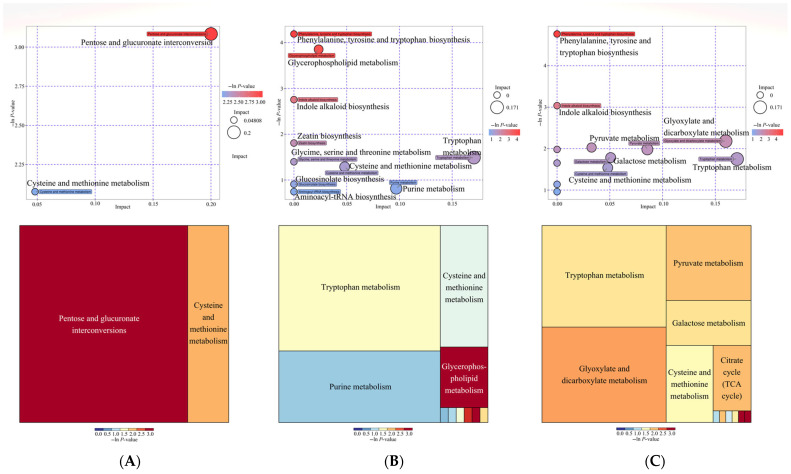
Bubble plots and tree plot of pathway analysis for the differential metabolites in group. (**A**) S1 vs. S2; (**B**) S1 vs. S3; (**C**) S1 vs. S4.

The KEGG database provides a reference knowledge network for linking metabolites from *C. vietnamensis* seeds in each development period to biological processes through PATHWAY mapping. In this study, the metabolic pathways enriched by differential metabolites were “pentose and glucuronate interconversions” and “cysteine and methionine metabolism” in Group S1 vs. S2; mainly “tryptophan metabolism”, “purine metabolism”, “cysteine and methionine metabolism”, and “glycerophospholipid metabolism” in Group S1 vs. S3; and in Group S1 vs. S4, they mainly were “tryptophan metabolism”, “glyoxylate and dicarboxylate metabolism”, “pyruvate metabolism”, “galactose metabolism”, “cysteine and methionine metabolism”, and the “citrate (TCA) cycle” (Figure 5).

After obtaining the matching information of each group of differentially expressed metabolites, we performed correlation network analysis on the KEGG database (Figure S4). The results showed that phloretin (flavonoid biosynthesis and circadian rhythm) and 5’-methylthioadenosine (MAPK signaling pathway and glycerophospholipid metabolism) were the differentially expressed metabolites when seeds were in the growth periods of S2 and S3, and indole (tryptophan metabolism) and L-tryptophan (tryptophan metabolism) were the differentially expressed metabolites when seeds were in the growth periods of S3 and S4 (Figure S5). In addition, flavonoid biosynthesis could be focused when camellia seeds in nutrition synthesis turned to fat accumulation, and the detailed information for this is displayed in Table S4.

### 2.6. Identification and Analysis of Flavonoids at Different Seed Maturation Stages

**Figure 6:**
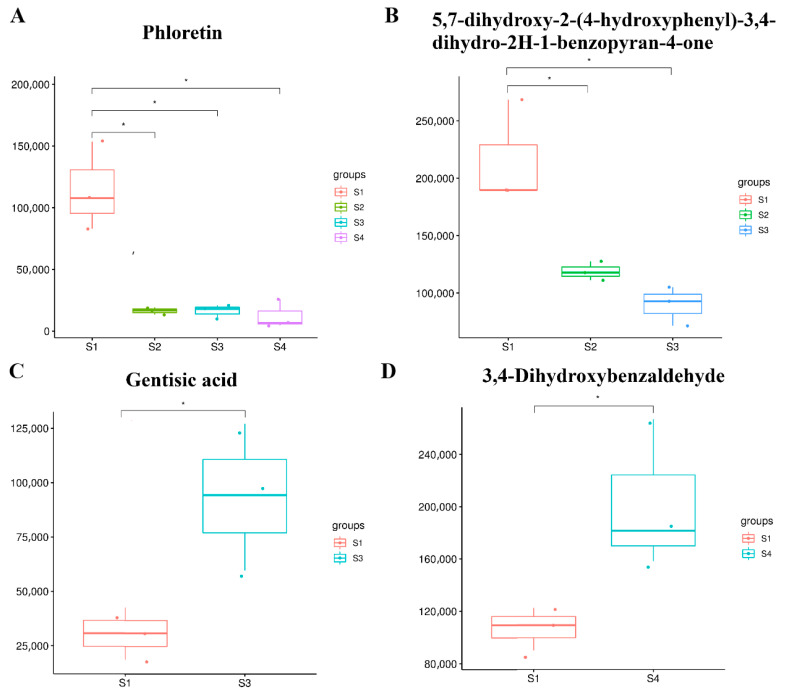
Box-and-whisker plots of differential metabolites related to flavonoids. (**A**) Phloretin; (**B**) 5,7-dihydroxy-2-(4-hydroxyphenyl)-3,4-dihydro-2H-1-benzopyran-4-one; (**C**) Gentisic acid; (**D**) 3,4-Dihydroxybenzaldehyde. * represented signifificance at level 0.05.

Flavonoids are an important class of metabolites in plants. In this context, 34 flavonoid metabolites were detected, of which 4 were differentially expressed (Tables S1 and S3). Phloretin was significantly down-regulated in all three comparison groups, and 5,7-dihydroxy-2-(4-hydroxyphenyl)-3, 4-dihydro-2H-1-benzopyran-4-one was notably down-regulated in both S1 vs. S2 and S1 vs. S3 (Figure 6A,B). Gentisic acid was found to be induced when oil-tea camellia seed was in the mature stage. In addition, 3,4-dihydroxybenzaldehyde in seeds increased in the late mature stage (Figure 6C,D). This indicates that flavonoids dynamically change during all the oil-tea camellia seed development stages, possibly being related to the nutrient synthesis or accumulation in seeds.

**Figure 7:**
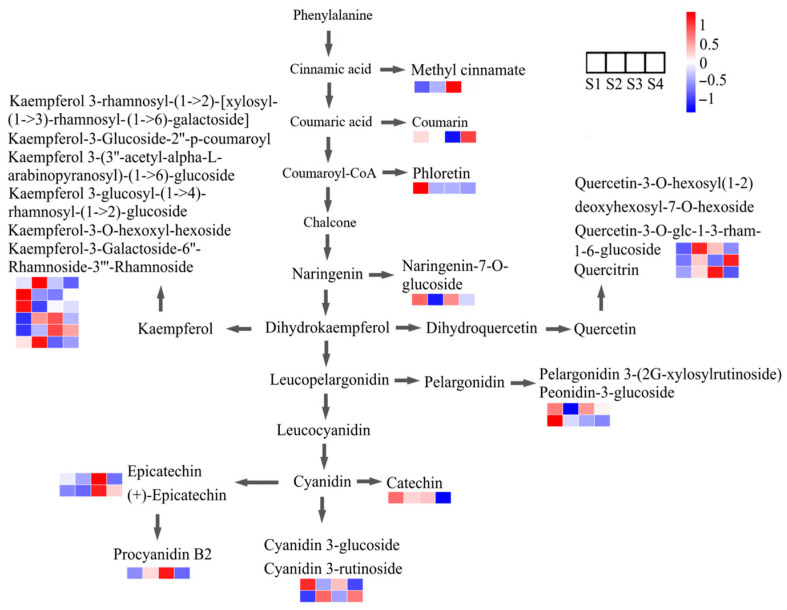
Metabolic pathways of flavonoids biosynthesis in *C. vietnamensis* seeds. Blue-white-red represented metabolites from down-regulated to upregulated.

The flavonoid biosynthesis pathway in *C. vietnamensis* seeds was further analyzed and mapped (Figure 7). The changes in the content of each metabolite in the flavonoid pathway can be clearly observed in Figure 7.

## 3. Discussion

### 3.1. General Features of Metabolites during Seed Development of C. Vietnamensis

In this paper, we performed an analysis of the metabolic components of seed samples in *C. vietnamensis* at different maturation stages (including nutrition synthesis, fat accumulation, mature, and late mature) and found 15, 32, and 30 significant differentially expressed metabolites in the S1 vs. S2, S1 vs. S3, and S1 vs. S4 comparison groups, respectively. They mainly included benzene and substituted derivatives, followed by linear 1,3-diarylpropanoids, purine nucleotides, glycerophospholipids, and a small number of indoles and derivatives and organooxygen compounds. The results from this study showed that metabolites classified as benzene and substituted derivatives ranked first among the differentially expressed metabolites. Aromatic compounds, such as poly-substituted benzene derivatives, play an important role in being antibacterial agents, optoelectronic materials, and chiral ligands, which have received special attention [20]. A component of 3-Methyl-1-(2,4,6-trihydroxyphenyl)-1-butanone was found to be preleptospermone. Dayan et al. explored a natural herbicide, manuka oil, and tested its primary component leptospermone, and its soil stability and bioavailability, providing a better understanding of the basis for pre-applied (PRE) activity of soil [21]. The reason those differential metabolites, such as 3-Methyl-1-(2,4,6-trihydroxyphenyl)-1-butanone, showed a down-regulated expressional trend during the seed ripening stages of *C. vietnamensis* may be related to the adaptation to environmental factors, including soil, during plant growth and reproduction.

### 3.2. Camellia Oil of the Fruit Ripening Period in C. vietnamensis

*Camellia* oil is a major nutritious substance stored in *C. vietnamensis* seeds. The oil content of dry seed was up to 42.01% in seed full maturity stage [5]. It has been suggested that a decrease in lipid content occurs at the final stage of the seed maturation process [22,23]. We also found that fatty acid content increased rapidly during seed development, peaked at S2, and then decreased gradually [6]. It might be that fatty acids start to decompose and be consumed in the fruit ripening period [24]. Our results showed that the content of kojibiose classified in fatty acyls in the late mature period was significantly higher than that in the nutrition synthesis stage. It is speculated that the fatty acids in the late mature period were decomposed to fatty acyls.

In this study, two compounds, including lysophosphatidylethanolamines (LysoPEs) (18:1(9Z)/0:0) and lysophosphatidlycholines (LysoPCs) (18:2(9Z,12Z)) were classified in glycerophospholipid metabolism. LysoPEs are hydrolyzed products of glycerophospholipids with phospholipase A1 (PLA1) and A2 (PLA2) [25], and phospholipid synthesis is definitely inhibited when cell injury or apoptosis occurs [26]. The increase in LysoPE (18:1(9Z)/0:0) levels in the mature periods of the seeds (S3 and S4) suggests that the oil-tea tree can improve glycerophospholipid metabolism disorder. In addition, previous studies have shown that LysoPCs are highly correlated with cell apoptosis, inflammatory, diet-induced hyperlipidemia, or even glucose regulation [27,28]. Lin et al. speculated three possible mechanisms of the incorporation of phosphatidylcholine-derived fatty acid in triacylglycerol and which one involved the transfer of the fatty acid from phosphatidylcholine to triacylglycerol to form LysoPCs and triacylglycerol [29]. Then, acyl-CoA:lysophosphatidylcholine acyltransferase (LPCAT) regenerated phosphatidylcholine from LysoPCs. The accumulation of camellia oil ought to reveal some essential biological processes, including phosphatidylcholine metabolism [29]. In our results, the LysoPC (18:2(9Z,12Z)) level increased in the mature stage of oil-tea tree seeds, which was consistent with the hypothesis of the relationship between fatty acids and LysoPCs.

### 3.3. Nutritional Components of the Fruit Ripening Period in C. vietnamensis

Some studies have shown that soluble sugar content is higher in the early development period in the plant’s seed but lower in seed oil synthesis and aging [30,31,32]. The results from the differential metabolite analysis showed that one compound (5’-Methylthioadenosine) classified in 5’-deoxyribonucleosides significantly degraded with oil-tea tree seed development. One beta-D-glucopyranoside level decreased in the mature stage (S3), while the D-xylulose level increased in the fat accumulation period compared to that in the nutrient accumulation stage, which might be due to the consumption of a large amount of carbohydrates and its conjugates for oil-tea tree fruit ripening and oil accumulation [24]. The increase in LysoPC (18:2(9Z,12Z)) content also confirmed Lin’s [33] research results that sucrose produced more defects where LysoPCs can insert, providing a better understanding of glucose regulation to LysoPCs.

Flavonoids, a natural polyphenol group in plants, widely exist in nature, with antioxidant, anti-inflammatory, anti-cancer, anti-tumor, protecting gastric mucosa, etc., functions [34,35], and are widespread in the seeds and flowers of *C. oleifera* [36,37]. The results from the differentially expressed metabolite analysis showed that the content of phloretin was significantly down-regulated during oil-tea tree fruit development, which was consistent with the finding in pomegranate (*Punica granatum* L.) [38] and apple (Malus × domestica) [39]. The higher content of gentisic acid in the mature stage of the seeds is interesting. As an important kind of phenolic acid, gentisic acid enables its conjugated form to have greater antioxidant efficacy than the free form, which becomes a material that can combine multiple natural bioactive compounds to intermeddle food waste and loss in food chemistry [40,41]. Another significantly increased flavonoid compound (3,4-dihydroxybenzaldehyde) in the mature stage of the seeds can weaken pentachlorophenol-induced cytotoxicity, DNA damage, and oxidative damage of human blood cell components and is able to function as a chemoprotective agent against pentachlorophenol or other harmful effects of chlorophenols [42]. Thus, the extracts from the ripe fruits of *C. vietnamensis*, especially high-level gentisic acid and the plant’s natural antioxidant 3,4-dihydroxybenzaldehyde could be used in the food industry to ensure the freshness of food and as medical agents. The flavonoid biosynthesis pathway is further mapped in Figure 7. As shown in Figure 7, cyanidin is a key intermediate for catechin, epicatechin, and cyanidin 3-glucoside synthesis. Phenylalanine is catalyzed by a series of enzymes such as ferulate-5-hydroxylase (*F5H*), dihydroflavonol 4-reductase (*DFR*), anthocyanidin reductase (*ANR*), leucoanthocyanidin reductase (*LAR*) to produce dihydrokaempferol, and then cyanidin [6,10]. It is also the common precursor of anthocyanins, flavonols, flavone glycosides, and so on [10]. In this pathway, the downstream metabolites epicatechin and procyanidin B2 were significantly higher in S3, while catechin and cyanidin 3-rutinoside showed a downward trend. The reason for this phenomenon might be that the expression of related genes (such as *F5H*, *DFR*, *LAR*, and *ANR*) had changed [6]. In addition, dihydroquercetin is a common precursor for both quercetin and leucocyanidin [10]. Consequently, it was reasonable that large amounts of cyanidin-based anthocyanins accumulated in *C. vietnamensis* seeds should enhance the production of quercetin. The widely enhanced flavonoid pathway could provide more available precursors for flavanol or anthocyanin biosynthesis in *C. vietnamensis* seeds.

Tea saponins, oleanane-type pentacyclic triterpenoids, have multiple biological activities, such as antimicrobial activity, strong contact toxicity, and stomach toxicity against pests [43], and are difficult to extract from *C. oleifera* seed [44]. The principal active ingredients of *C. oleifera* seed are saponins, including 77 kinds saponins with different structures (camelliasaponin, theasaponin E1/E2, etc.), which are widely used in cooking or in the medical agent industry [43,45]. One β-amyrin (precursor in the upstream of tea saponin synthesis) and five tea saponin monomers ((2alpha,3beta,5xi,9xi,18xi)-2,3-Dihydroxyolean-12-en-28-oic acid; Olean-12-en-28-oic acid, 2,3,19,24-tetrahydroxy-, (2alpha,3beta,5xi,9xi,19alpha)-; 1-O-[(3beta,5xi,9xi,18xi)-3-(beta-D-Glucopyranuronosyloxy)-28-oxoolean-12-en-28-yl]-beta-D-glucopyranose; (3beta,5xi,9xi,16beta,18xi,21beta,22alpha)-28-Acetoxy-16,22,23-trihydroxy-21-[(2-methylbutanoyl)oxy]olean-12-en-3-yl beta-D-glucopyranosiduronic acid; and Olean-12-en-28-oic acid, 3-hydroxy-, (3beta,5xi,9xi,18xi)-) were detected in this metabolic profiling. Although their content maintained a relatively stable trend throughout the development of *C. vietnamensis* seeds, the change trend between the different substances was slightly different. The content of β-amyrin and three saponins (Olean-12-en-28-oic acid, 2,3,19,24-tetrahydroxy-, (2alpha,3beta,5xi,9xi,19alpha)-; 1-O-[(3beta,5xi,9xi,18xi)-3-(beta-D-Glucopyranuronosyloxy)-28-oxoolean-12-en-28-yl]-beta-D-glucopyranose; and Olean-12-en-28-oic acid, 3-hydroxy-, (3beta,5xi,9xi,18xi)-) reached the highest in the S2 period, while the content of (2alpha,3beta,5xi,9xi,18xi)-2,3-Dihydroxyolean-12-en-28-oic acid and (3beta,5xi,9xi,16beta,18xi,21beta,22alpha)-28-Acetoxy-16,22,23-trihydroxy-21-[(2-methylbutanoyl)oxy]olean-12-en-3-yl beta-D-glucopyranosiduronic acid were the highest in the S3 period. The results indicated that the seeds had an abundance of saponins, which might lead to the bitterness of *Camellia* oil.

## 4. Materials and Methods

### 4.1. Plant Materials

Fresh *C. vietnamensis* fruits at the full maturity stage that were uniform in size, shape, and color, and that had not been attacked by insects, were collected from Yangjiang town (19°120′10″ N; 110°240′32″ E), Qionghai city, Hainan Province in China, where they had ample light exposure, a hot and rainy climate, and sandy red soil with pH 5.5, on 24 November 2018. The seeds of *C. vietnamensis* were harvested during the 2018 season at four different developmental periods: nutrition synthesis stage (S1, 24 August), fat accumulation stage (S2, 24 September), mature stage (S3, 24 October), and late mature stage (S4, 24 November) (Figure S1). Then, a total of four stages of seed samples were quickly placed into liquid nitrogen and then stored at −80 °C for metabolomic analysis (3 biological samples for each stage).

### 4.2. Sample Preparation

The freeze-dried samples were crushed with a mixer mill for 2 min at 60 Hz. Then, 100 mg powder of each sample was transferred to a 2 mL EP tube and extracted with 1500 μL methanol/water mixture (v:v = 3:1). The samples were vortexed for 30 s and ultra-sonicated for 15 min in an ice bath, followed by overnight shaking at 4 °C. Then, all the samples were centrifuged at 12,000 rpm for 15 min at 4 °C. The resulting supernatants were transferred to 2 mL glass vials and stored at −80 °C until the UHPLC/Q-TOF-MS analysis. The quality control (QC) sample was prepared by the mixing of an equal aliquot of the supernatants from all the samples [46].

### 4.3. UHPLC/Q-TOF-MS Analysis

The UHPLC separation was carried out using a Waters ACQUITY UPLC HSS T3 column (100 × 2.1 mm, 1.8 μm). Mobile phase A was 0.1% formic acid in water, and mobile phase B was acetonitrile. The gradient elution procedure was as follows: 0–0.5 min, 98% A, 2% B; 0.5–10 min, 50% A, 50% B; 10–11 min, 5% A, 95% B; 11–13 min, 5% A, 95% B; 13–15 min, 98% A, 2% B. The column temperature was set at 40 °C. The auto-sampler temperature was set at 4 °C and the injection volume was 2 μL [47,48].

An AB Sciex QTOF mass spectrometer was used for its ability to acquire MS/MS spectra using information-dependent acquisition (IDA) during an LC/MS experiment. In this mode, the acquisition software (Analyst) continuously evaluates the full scan survey MS data as it collects and triggers the acquisition of MS/MS spectra depending on preselected criteria. In each cycle, 5 precursor ions whose intensity was greater than 100 were chosen for fragmentation using collision energy. The acquired mass ranges were divided into 100–300, 300–450, 450–600, 600–750, and 750–1200 with 5 injections. The ESI source conditions were set as follows: the ion spray voltage was +5500/−4500 V, the curtain of gas was 35 psi, the temperature was 600 °C, the ion source of Gases 1 and 2 was both 60 psi, and the DP was ±100 V [47,48].

An AB Sciex QTrap 6500 mass spectrometer was used for assay development. The ion source parameters were: ion spray voltage of +5000/−4500 V, curtain of gas of 35 psi, temperature of 400 °C, ion source of Gases 1 and 2 of 60 psi, and DP of ±100 V.

### 4.4. Data Preprocessing and Annotation

The high-resolution MS data were converted to the mzXML format using ProteoWizard and processed using MAPS software (version 1.0, Dalian, China). The preprocessing results generated a data matrix that consisted of the retention time (RT), mass-to-charge ratio (*m*/*z*) values, and peak intensity. An in-house MS2 database was used for the metabolite identification. In addition, the MRM data were processed with Skyline software [49].

## 5. Conclusions

In the present study, a total of 293 metabolites were identified from the methanol extract of the seeds of *C. vietnamensis* by UHPLC/Q-TOF-MS analysis. Five metabolites, belonging to benzene and substituted derivatives, 5′-deoxyribonucleosides and linear 1,3-diarylpropanoids, were found in all three comparison groups, with consistent down-regulated trends. The KEGG results showed that phloretin and 5′-methylthioadenosine were the differentially expressed metabolites when seeds were in the growth periods of S2 and S3, and indole and L-tryptophan were the differentially expressed metabolites when seeds were in the growth periods of S3 and S4. In addition, 34 flavonoid metabolites were detected, of which 4 were differentially expressed. This indicated that the flavonoids dynamically change during all the oil-tea camellia seed development stages, possibly being related to the nutrient synthesis or accumulation in the seeds, which needs to be further studied. The results in this study showed that *C. vietnamensis* seeds should be collected in S2 to obtain a higher concentration of β-amyrin and in S3 or S4 to obtain a higher concentration of phenolic compounds (such as gentisic acid and 3,4-dihydroxybenzaldehyde).

## Data Availability

The data presented in this study are available on request from the corresponding author. The data are not publicly available due to privacy.

## Supplementary Materials

The following supporting information can be downloaded at: https://www.mdpi.com/article/10.3390/molecules27206817/s1, Figure S1: Phenotypic characteristics of seeds of *C. vietnamensis* in different maturation stages. S1–4, nutrition synthesis stage, fat accumulation stage, mature stage and late mature stage; Figure S2: OPLS-DA models of seeds of *C. vietnamensis* at different maturity stages in positive and negative ion modes; Figure S3: OPLS-DA permutation plot of seeds of *C. vietnamensis* at different maturity stages. A: S2 vs. S1, B: S3 vs. S1, C: S4 vs. S1; Figure S4: Correlation network analysis of differentially expressed metabolites in comparison groups. A: S1 vs. S2, B: S1 vs. S3, C: S1 vs. S4; Figure S5. Heatmap of differentially expressed metabolites in comparison groups. A: S1 vs. S2, B: S1 vs. S3, C: S1 vs. S4. Table S1: Detection of total metabolites using UHPLC-QTOFMS; Table S2: Metabolites in clusters; Table S3: Differentially Expressed Metabolites; Table S4: Regulatory network information of differentally expressed metabolites in seeds from *C. vietnamensis* at different maturity stages.

## References

[1] Feng J.L., Yang Z.J., Chen S.P., El-Kassaby Y.A., Chen H. (2017). High throughput sequencing of small RNAs reveals dynamic microRNAs expression of lipid metabolism during *Camellia oleifera* and *C. meiocarpa* seed natural drying. BMC Genom..

[2] Jin X.C. (2012). Bioactivities of water-soluble polysaccharides from fruit shell of *Camellia oleifera* Abel: Antitumor and antioxidant activities. Carbohyd. Polym..

[3] Zhu G.F., Liu H., Xie Y.C., Liao Q., Lin Y.W., Liu Y.H., Liu Y.H., Xiao H.W., Cao Z.J., Hu S.Z. (2020). Postharvest Processing and Storage Methods for *Camellia oleifera* Seeds. Food Rev. Int..

[4] Othman L., Sleiman A., Abdel-Massih R.M. (2019). Antimicrobial activity of polyphenols and alkaloids in middle eastern plants. Front. Microbiol..

[5] Ye Z.C., Wu Y.G., Ul Haq Muhammad Z., Yan W.P., Yu J., Zhang J.F., Yao G.L., Hu X.W. (2020). Complementary transcriptome and proteome profiling in the mature seeds of *Camellia oleifera* from Hainan Island. PLoS ONE.

[6] Ye Z.C., Yu J., Yan W.P., Zhang J.F., Yang D.M., Yao G.L., Liu Z.J., Wu Y.G., Hou X.L. (2021). Integrative iTRAQ-based proteomic and transcriptomic analysis reveals the accumulation patterns of key metabolites associated with oil quality during seed ripening of *Camellia oleifera*. Hortic. Res.-Engl..

[7] Chen J., Guo Y.J., Hu X.W., Zhou K.B. (2022). Comparison of the chloroplast genome sequences of 13 oil-tea *Camellia* samples and identification of an undetermined oil-tea *Camellia* species from Hainan province. Front. Plant Sci..

[8] Dai J.N., Zheng W., Yu J., Yan H.Q., Wang Y., Wu Y.G., Hu X.W., Lai H.G. (2022). cDNA cloning, prokaryotic expression, and functional analysis of squalene synthase (*SQS*) in *Camellia vietnamensis* Huang. Protein Express Purif..

[9] Jing J., Shi Y., Zhang Q., Wang J., Ruan J. (2017). Prediction of Chinese green tea ranking by metabolite profiling using ultra-performance liquid chromatography–quadrupole time-of-flight mass spectrometry (UPLC–Q-TOF/MS). Food Chem..

[10] Li M., Shen Y., Ling T., Ho C.-T., Li D., Guo H., Xie Z. (2021). Analysis of Differentiated Chemical Components between Zijuan Purple Tea and Yunkang Green Tea by UHPLC-Orbitrap-MS/MS Combined with Chemometrics. Foods.

[11] Deng W.W., Han J.Y., Fan Y.B., Tai Y.L., Zhu B.Y., Lu M.Q., Wang R.J., Wan X.C., Zhang Z.Z. (2018). Uncovering tea-specific secondary metabolism using transcriptomic and metabolomic analyses in grafts of *Camellia sinensis* and *C. oleifera*. Tree Genet. Genom..

[12] Chen S., Liu H., Zhao X., Li X., Shan W., Wang X., Wang S., Yu W., Yang Z., Yu X. (2020). Non-targeted metabolomics analysis reveals dynamic changes of volatile and non-volatile metabolites during oolong tea manufacture. Food Res. Int..

[13] Wu H.L., Huang W.J., Chen Z.J., Chen Z., Shi J.F., Kong Q., Sun S.L., Jiang X.H., Chen D., Yan S.J. (2019). GC-MS-based metabolomic study reveals dynamic changes of chemical compositions during black tea processing. Food Res. Int..

[14] Wang M.Y., Wang Q.L., Yang Q., Yan X.X., Feng S.X., Wang Z.N. (2020). Comparison of Anthraquinones, Iridoid Glycosides and Triterpenoids in *Morinda officinalis* and *Morinda citrifolia* Using UPLC/Q-TOF-MS and Multivariate Statistical Analysis. Molecules.

[15] Zhang L., Shen H., Xu J., Xu J.D., Li Z.L., Wu J., Zou Y.T., Liu L.F., Li S.L. (2018). UPLC-QTOF-MS/MS-guided isolation and purification of sulfur-containing derivatives from sulfur-fumigated edible herbs, a case study on ginseng. Food Chem..

[16] Lee J., Hwang Y.S., Chang W.S., Moon J.K., Choung M.G. (2013). Seed maturity differentially mediates metabolic responses in black soybean. Food Chem..

[17] Liu G., Chen H.P., Wu Z.H., Peng Y., Xie Y.J. (2019). Analyses of seed development of *Plukenetia volubilis* by joint metabolomics and transcriptomics approaches. Sci. Silvae Sin..

[18] Yang C., Wu P., Yao X., Sheng Y., Zhang C., Lin P., Wang K. (2022). Integrated Transcriptome and Metabolome Analysis Reveals Key Metabolites Involved in *Camellia oleifera* Defense against Anthracnose. Int. J. Mol. Sci..

[19] He Y., Chen R., Yang Y., Liang G., Zhang H., Deng X., Xi R. (2022). Sugar Metabolism and Transcriptome Analysis Reveal Key Sugar Transporters during *Camellia oleifera* Fruit Development. Int. J. Mol. Sci..

[20] Zhang H., Zhang X., Chen X., Qu H.M. (2018). Synthesis mediated by zirconium, crystal structure and theoretical studies on six-substituted benzene with silalactone structure and butadiene derivative. Chin. J. Struct. Chem..

[21] Dayan F., Howell J., Marais J., Ferreira D., Koivunen M. (2011). Manuka Oil, A Natural Herbicide with Preemergence Activity. Weed Sci..

[22] Baud S., Lepiniec L. (2009). Regulation of de novo fatty acid synthesis in maturing oilseeds of *Arabidopsis*. Plant Physiol. Biochem..

[23] Peng S.F., Lu J., Zhang Z., Ma L., Liu C.X., Chen Y.Z. (2020). Global transcriptome and correlation analysis reveal cultivar-specific molecular signatures associated with fruit development and fatty acid determination in *Camellia oleifera* Abel. Int. J. Genom..

[24] Iqbal N., Khan N.A., Ferrante A., Trivellini A., Francini A., Khan M.I.R. (2017). Ethylene Role in Plant Growth, Development and Senescence: Interaction with Other Phytohormones. Front. Plant Sci..

[25] Yamamoto Y., Sakurai T., Chen Z., Furukawa T., Gowda S.G.B., Wu Y., Nouso K., Fujii Y., Yoshikawa Y., Chiba H. (2021). Analysis of serum lysophosphatidylethanolamine levels in patients with non-alcoholic fatty liver disease by liquid chromatography-tandem mass spectrometry. Anal. Bioanal. Chem..

[26] Jiang Y., Qu K., Liu J.C., Wen Y., Duan B.H. (2022). Metabolomics study on liver of db/db mice treated with curcumin using UPLC-Q-TOF-MS. J. Pharm. Biomed..

[27] Liu L.Y., Yin T.L., Chen Y., Li Y.H., Yin L., Ding J.L., Yang J., Feng H.-L. (2019). Follicular dynamics of glycerophospholipid and sphingolipid metabolisms in polycystic ovary syndrome patients. J. Steroid Biochem..

[28] Miao H., Chen H., Pei S.W., Bai X., Vaziri N.D., Zhao Y.Y. (2015). Plasma lipidomics reveal profound perturbation of glycerophospholipids, fatty acids, and sphingolipids in diet-induced hyperlipidemia. Chem.-Biol. Interact..

[29] Lin P., Wang K., Zhou C., Xie Y., Yao X., Yin H. (2018). Seed transcriptomics analysis in *Camellia oleifera* uncovers genes associated with oil content and fatty acid composition. Int. J. Mol. Sci..

[30] Baud S., Boutin J.P., Miquel M., Lepiniec L., Rochat C. (2002). An integrated overview of seed development in *Arabidopsis thaliana* ecotype WS. Plant Physiol. Biochem..

[31] Bvttner M. (2010). The *Arabidopsis* sugar transporter (AtSTP) family: An update. Plant Biol..

[32] Periappuram C., Steinhauer L., Barton D.L., Taylor D.C., Chatson B., Zou J.T. (2000). The plastidic phosphoglucomutase from arabidopsis. A reversible enzyme reaction with an important role in metabolic control. Plant Physiol..

[33] Diaz S.B., Lopez de Biondi A.C., Disalvo E.A. (2003). Dehydration of carbonyls and phosphates of phosphatidylcholines determines the lytic action of lysoderivatives. Chem. Phys. Lipids.

[34] Liu D.M., Yu X., Sun H.Y., Zhang W., Liu G., Zhu L. (2020). Flos lonicerae flavonoids attenuate experimental ulcerative colitis in rats via suppression of NF-κB signaling pathway. Naunyn-Schmiedeberg’s Arch. Pharmacol..

[35] Zhang C.Y., Li X.Q., Jin S.L., Zhang H.Z., Wang R., Pei L.H., Zheng J. (2020). The anti-proliferative effect of flavonoid nanoparticles on the human ovarian cancer cell line SK0V3. J. Nanosci. Nanotechnol..

[36] Liu X.H., Jia L.Y., Gao Y., Li B., Tu Y.Y. (2014). Anti-inflammatory activity of total flavonoids from seeds of *Camellia oleifera* Abel. Acta Biochim. Biophys. Sin..

[37] Ma Y.Q., Liu M.H., Tan T., Yan A.P., Guo L., Jiang K., Tan C.H., Wan Y.Q. (2018). Deep eutectic solvents used as extraction solvent for the determination of flavonoids from *Camellia oleifera* flowers by high-performance liquid chromatography. Phytochem. Anal..

[38] Han L.L., Yuan Z.H., Feng L.J., Yin Y. (2015). Changes in the composition and contents of pomegranate polyphenols during fruit development. Acta Hortic..

[39] Dare A.P., Yauk Y.K., Tomes S., Mcghie T.K., Rebstock R.S., Rebstock R.S., Atkinson R.G. (2017). Silencing a phloretin-specific glycosyltransferase perturbsboth general phenylpropanoid biosynthesis and plant development. Plant J. Cell Mol. Biol..

[40] Li H., Pan Y.X., Yang Z.Y., Rao J., Chen B. (2019). Improving antioxidant activity of β-lactoglobulin by nature-inspired conjugation with gentisic acid. J. Agric. Food Chem..

[41] Li H., Pan Y.X., Li C., Yang Z.Y., Rao J.J., Chen B.C. (2022). Design, synthesis and characterization of lysozyme–gentisic acid dual-functional conjugates with antibacterial/antioxidant activities. Food Chem..

[42] Maheshwari N., Mahmood R. (2022). 3,4-Dihydroxybenzaldehyde attenuates pentachlorophenol-induced cytotoxicity, DNA damage and collapse of mitochondrial membrane potential in isolated human blood cells. Drug Chem. Toxicol..

[43] Guo N., Tong T.T., Ren N., Tu Y.Y., Li B. (2018). Saponins from seeds of Genus *Camellia*: Phytochemistry and bioactivity. Phytochemistry.

[44] Zhang S.Y., Pan Y.G., Zheng L.L., Yang Y., Zheng X.Y., Ai B.L., Xu Z.M., Sheng Z.W. (2019). Application of steam explosion in oil extraction of camellia seed (*Camellia oleifera* Abel.) and evaluation of its physicochemical properties, fatty acid, and antioxidant activities. Food Sci. Nutr..

[45] Chen Y.-F., Yang C.-H., Chang M.-S., Ciou Y.-P., Huang Y.-C. (2010). Foam Properties and Detergent Abilities of the Saponins from *Camellia oleifera*. Int. J. Mol. Sci..

[46] Chen W., Gong L., Guo Z.L., Wang W.S., Zhang H.Y., Liu X.Q., Yu S.B., Xiong L.Z., Luo J. (2013). A Novel integrated method for large-scale detection, identification, and quantification of widely targeted metabolites: Application in the study of rice metabolomics. Mol. Plant.

[47] Luo P., Yin P.Y., Zhang W.J., Zhou L.N., Lu X., Lin X.H., Xu G.W. (2016). Optimization of large-scale pseudotargeted metabolomics method based on liquid chromatography-mass spectrometry. J. Chromatogr. A.

[48] Zha H.B., Cai Y.P., Yin Y.D., Wang Z.Z., Li K., Zhu Z.J. (2018). SWATH to MRM: Development of high-coverage targeted metabolomics method using SWATH technology for biomarker discovery. Anal. Chem..

[49] Kuhl C., Tautenhahn R., Böttcher C., Larson T.R., Neumann S. (2012). CAMERA: An integrated strategy for compound spectra extraction and annotation of liquid chromatography/mass spectrometry data sets. Anal. Chem..

