# Supplementary material for: Comparison of the Main Metabolites in Different Maturation Stages of Camellia vietnamensis Huang Seeds
Source: Molecules. 2022 Oct 12;27(20):6817. doi: 10.3390/molecules27206817 (PMC9608468; doi:10.3390/molecules27206817)
Supplement: Supplementary file 1 [file molecules-27-06817-s001.zip › Supplementary figures.pdf]

Attached Figures

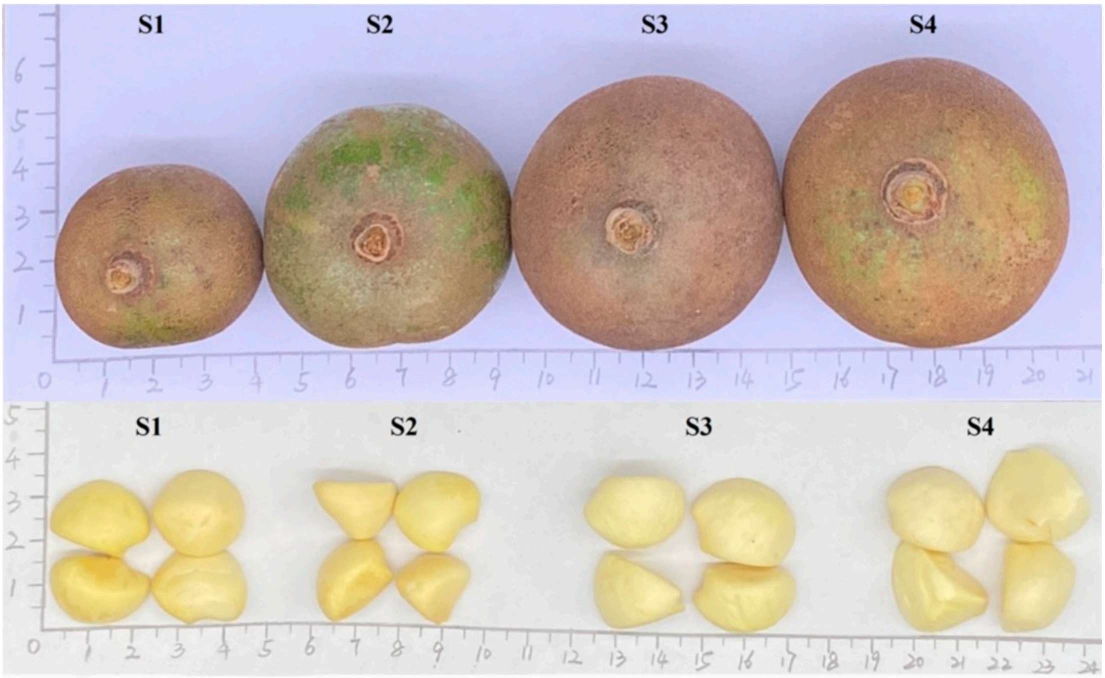

Figure S1. Phenotypic characteristics of seeds of *C. vietnamensis* in different maturation stages. S1-4, nutrition synthesis stage, fat accumulation stage, mature stage and late mature stage (Ye et al., 2021).

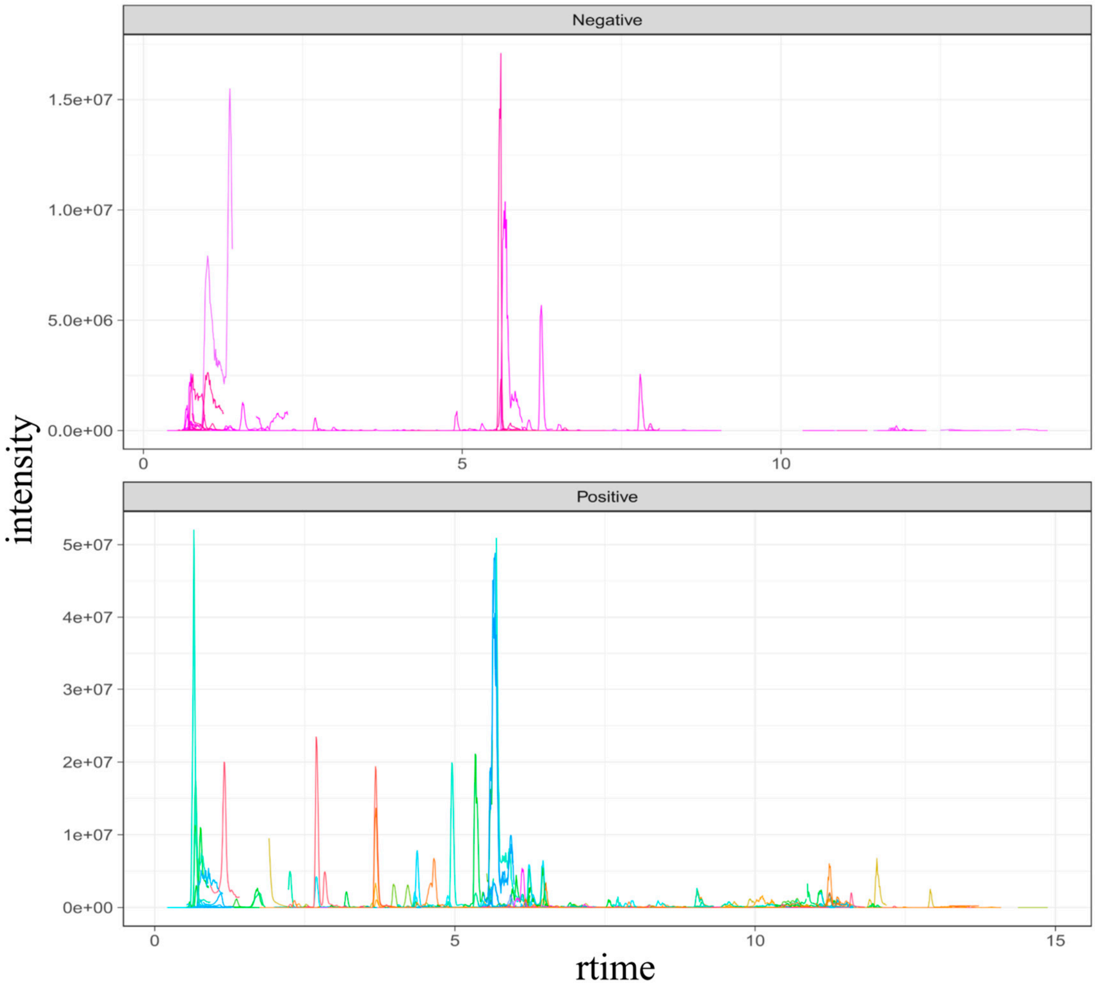

Figure S2. The total ion chromatography (TIC) of QC in negative and positive iron modes.

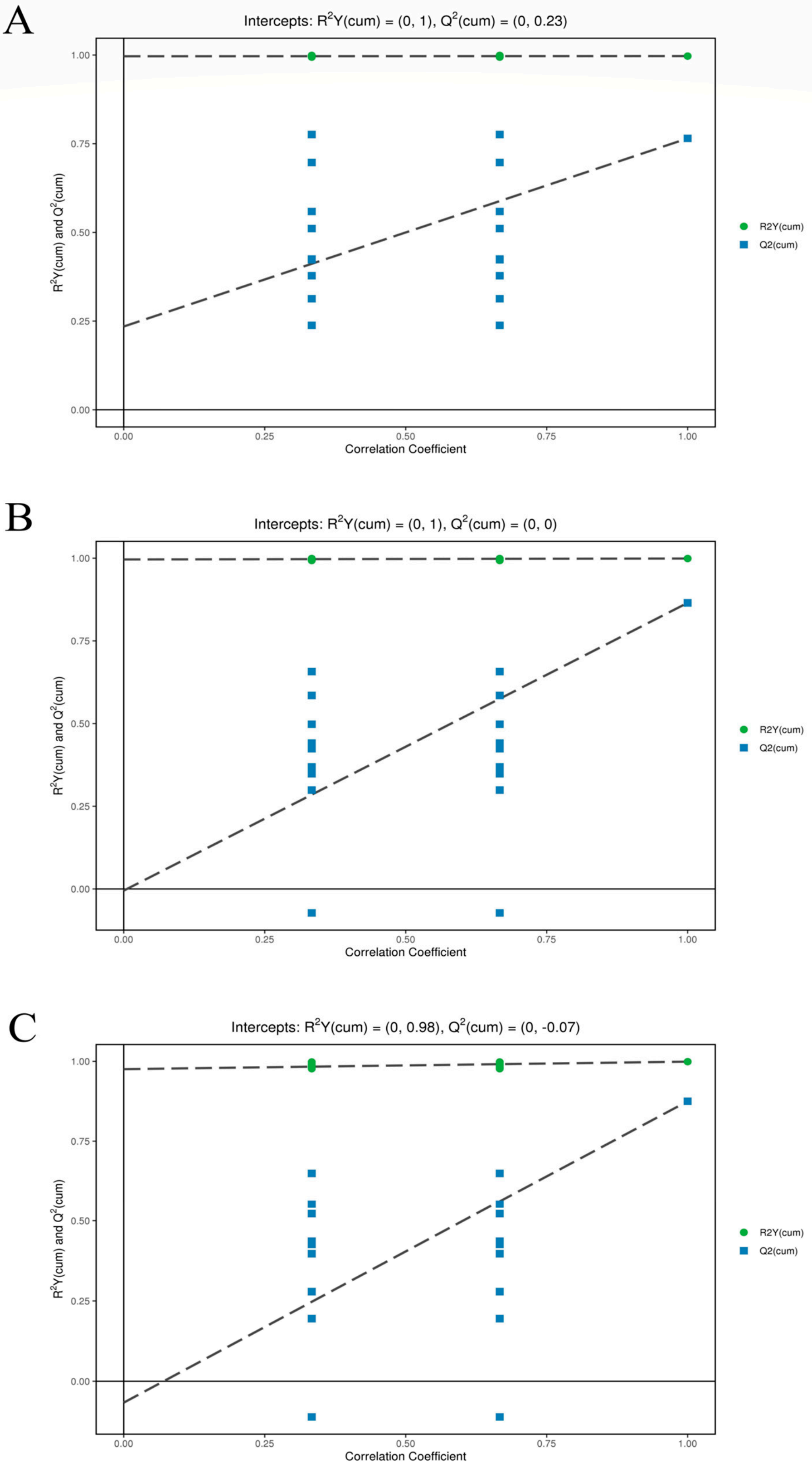

Figure S3. OPLS-DA permutation plot of seeds of *C. oleifera* at different maturity stages. A: S2 vs. S1, B: S3 vs. S1, C: S4 vs. S1.

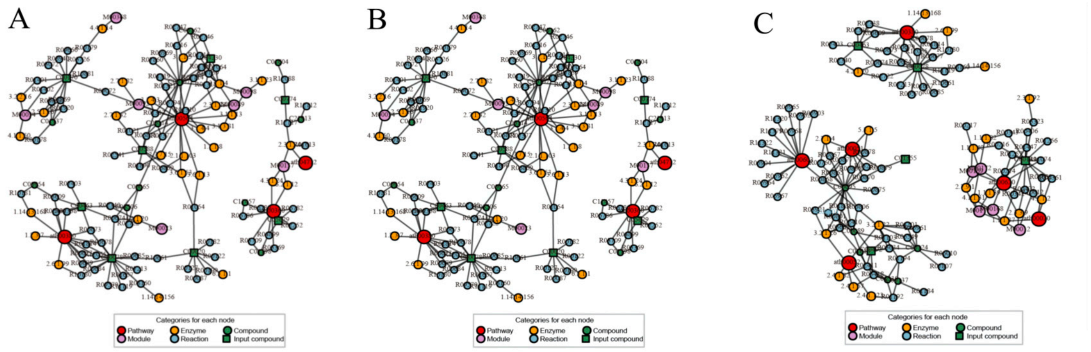

Figure S4. Correlation network analysis of differentially expressed metabolites in comparison groups. A: S1 vs. S2, B: S1 vs. S3, C: S1 vs. S4.

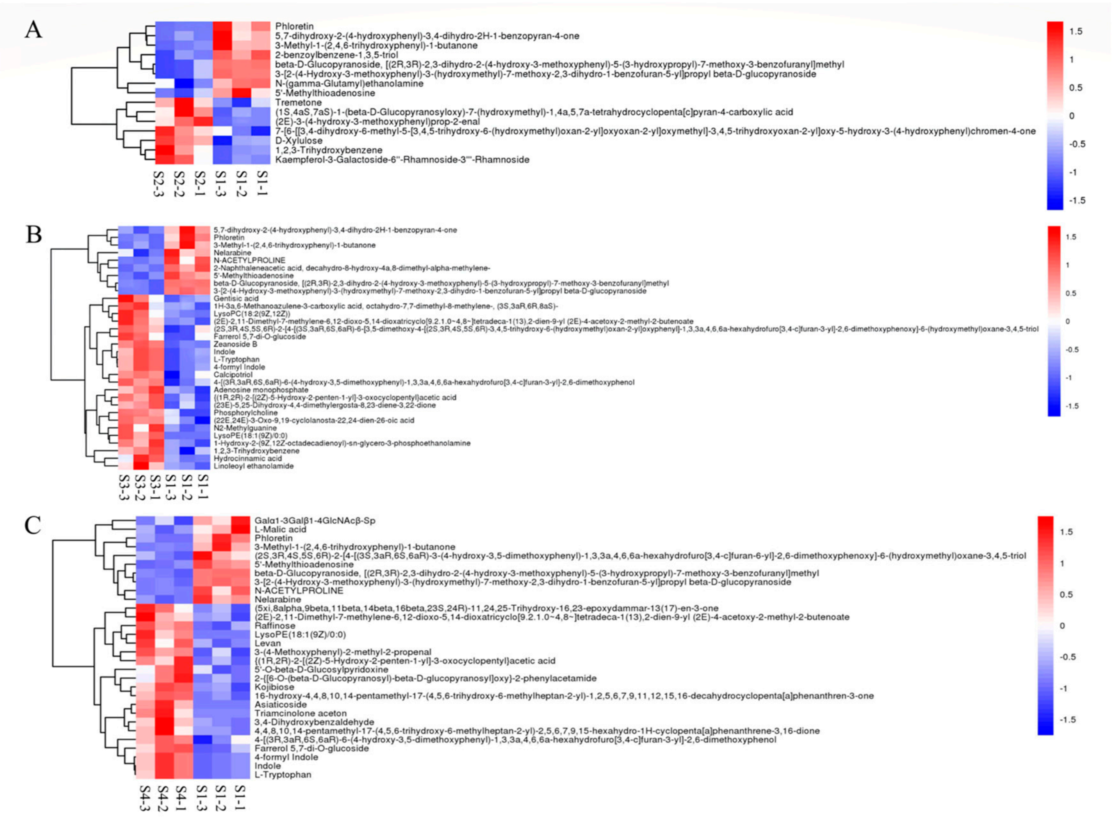

Figure S5. Heatmap of differentially expressed metabolites in comparison groups. A: S1 vs. S2, B: S1 vs. S3, C: S1 vs. S4.
